# Current News

**Published:** 1897-06

**Authors:** 


### Current News.

### RESOLUTION OF THE ODONTOLOGICAL SOCIETY OF PENNSYLVANIA.

A PAMPHLET having been issued purporting to be a report of a committee made at a special meeting of the Odontological Society of Pennsylvania, held November 1896, the publication and distribution of this said report being wholly unauthorized by the society before which it was read, the following resolution was at the Annual Meeting held May 8, 1897, unanimously adopted:

*Resolved*, That the Editor of the Proceedings, Dr. Jos. Head, be and is hereby authorized to publish in the *Dental Cosmos* and INTERNATIONAL DENTAL JOURNAL (if possible) the statement that the Odontological Society of Pennsylvania authorized neither the publication nor the distribution of this said pamphlet, which purports to be a reprint from the Proceedings of the Odontological Society of Pennsylvania, November, 1896.

C. N. PEIRCE.

### PENNSYLVANIA STATE DENTAL EXAMINING BOARD.

THE Pennsylvania State Dental Examining Board will meet at the Glen Summit House, Glen Summit, Luzerne County, Pa., on Tuesday, July 6, 1897.

HENRY GERHART, Lewisburg, Pa.,
*President.*

J. C. GREEN, West Chester, Pa.,
*Secretary.*

### PENNSYLVANIA STATE DENTAL SOCIETY.

THE Twenty-ninth Annual Meeting of the Pennsylvania State Dental Society will be held at Glen Summit, Pa., on July 6, 7, and 8, 1897.

A full and interesting programme has been arranged and a cordial invitation is extended to all. Ticket-orders for reduced rates can be had over the following roads: Pennsylvania, Philadelphia and Reading, Lehigh Valley, and Central Railroad of New Jersey, by stating over which route you wish to travel and addressing

DR. E. S. JONES, Bethlehem, Pa.,
*Corresponding Secretary.*

