# Correction

**Published:** 1897-07

**Authors:** L. Ashley Faught

**Affiliations:** Philadelphia, Pa.


### CORRECTION.

PHILADELPHIA, PA., June 7, 1897.

TO THE EDITOR:

SIR,—There appears in the June issue of your journal, over the signature of Dr. C. N. Peirce, what appears to be a resolution adopted by the Odontological Society, at its May meeting. As I originally framed and presented the resolution adopted, I wish to state that its language as now printed by you is incorrectly worded and misleading in character. The resolution which I presented and which was adopted is as follows:

*Resolved*, That this Association request the editor (Dr. Joseph Head) to publish in the *Dental Cosmos* and in the INTERNATIONAL DENTAL JOURNAL a disclaimer of the responsibility of this Association for the issuing of the reprints of the proceedings of its special meeting, held on November 18, 1897.

In the interest of truth and fairness, may I not request that you either publish this letter or make the correction in the next issue of your journal.

Sincerely yours,
L. ASHLEY FAUGHT.

